# ARID1A regulates histone octamer transfer activity of human canonical BAF complex

**DOI:** 10.1093/nar/gkaf958

**Published:** 2025-09-29

**Authors:** Naoe Moro, Yukiko Fujisawa-Tanaka, Shinya Watanabe

**Affiliations:** Program in Molecular Medicine, University of Massachusetts Chan Medical School, Worcester, MA 01605, United States; Program in Molecular Medicine, University of Massachusetts Chan Medical School, Worcester, MA 01605, United States; Program in Molecular Medicine, University of Massachusetts Chan Medical School, Worcester, MA 01605, United States

## Abstract

Mutations that impact subunits of mammalian SWI/SNF (mSWI/SNF or BAF) chromatin remodeling complexes are found in over 20% of human cancers. Among these subunits, AT-rich interactive domain-containing protein 1A (ARID1A) is the most frequently mutated gene, occurring in over 8% of various cancers. The majority of ARID1A mutations are frameshift or nonsense mutations, causing loss of function. Previous studies have suggested that ARID1A may facilitate interactions between BAF complexes and various transcriptional coactivators, but a biochemical role for ARID1A in BAF remodeling activity has not been identified. Here, we describe the *in vitro* reconstitution of the cBAF, PBAF, and ncBAF complexes, and we compare their biochemical activities. In addition, we reconstitute a variety of cBAF subcomplexes, defining roles for several subunits in high affinity nucleosome binding and nucleosome sliding activity. Remarkably, we find that the ARID1A subunit of cBAF is largely dispensable for nucleosome binding, nucleosome sliding, and adenosine triphosphatase activity, but ARID1A is required for cBAF to transfer histone octamers between DNA templates. Our study reveals a biochemical function of ARID1A/ARID1B in BAF-mediated chromatin remodeling, suggesting a model in which dysregulation of histone octamer transfer activity of BAF complexes may be relevant to cancer formation.

## Graphical Abstract

**Graphical Abstract:**
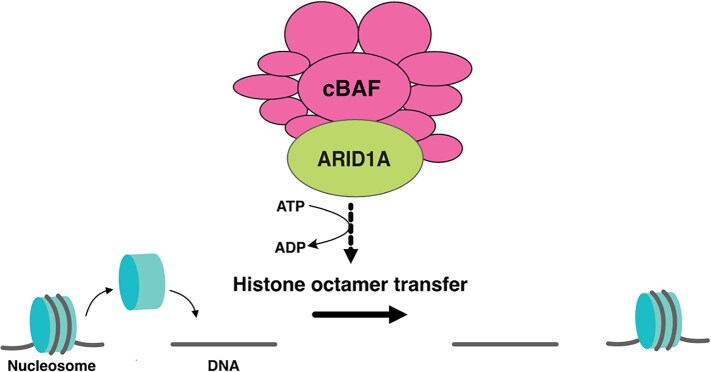

## Introduction

Adenosine triphosphate (ATP)-dependent chromatin remodeling enzymes are involved in almost all DNA metabolic processes, such as transcription, DNA repair, and DNA replication. They utilize the energy from ATP hydrolysis to directly alter chromatin structure by catalyzing the sliding of histone octamers *in cis* along DNA, the transfer or exchange of histone H2A/H2B dimers, or the ejection or transfer of an entire histone octamer [1]. How each of these distinct biochemical activities contributes to a specific cellular function is not clear since inactivation of a key remodeling subunit typically inactivates all activities of a remodeling enzyme.

Genes encoding subunits of mammalian SWI/SNF (mSWI/SNF or BAF) chromatin remodeling complexes are mutated in over 20% of human cancers [2, 3]. Mutations impacting BAF subunits also cause neurodevelopment disorders, such as Coffin–Siris syndrome [4]. Human BAF complexes exist as three major complexes—cBAF (canonical BRG1/BRM-associated factors), PBAF (polybromo-associated BAF), and ncBAF (noncanonical BAF), which differ based on their distinct subunit compositions. To date, the cBAF complex is believed to be composed of 12 subunits, 13 subunits for PBAF, and 10 subunits for the ncBAF complex. Several BAF subunits also have multiple isoforms, extending the diversity of BAF complex composition. CryoEM analyses of cBAF and PBAF indicate that these complexes consist of three modules—an adenosine triphosphatase (ATPase) module, an ARP module, and a Base module [5, 6]. The ATPase module is composed of the ATPase domain of the BRG1 (SMARCA4) or BRM (SMARCA2) subunit. The ARP module contains *ß*-actin and BAF53a (ACTL6A) that are associated with the N-terminal HSA domain of BRG1, and the ARP module hinges the ATPase and Base modules. The Base module consists of five core subunits shared among both cBAF and PBAF complexes [BAF170 (SMARCC2), BAF155 (SMARCC1), BAF60 (SMARCD1), BAF57 (SMARCE1), and BAF47 (SMARCB1)], as well as cBAF- or PBAF-specific subunits. cBAF and PBAF engage the nucleosome by sandwiching it between the ATPase module and the BAF47 (SMARCB1) subunit within the Base module.

The AT-rich interactive domain-containing protein 1A (ARID1A) is a cBAF-specific subunit and a component of the Base module. ARID1A is the most frequently mutated gene among BAF subunits (∼8% of all types of cancer) [7]. In gynecologic cancers, ARID1A mutations are found in 60% of ovarian clear-cell carcinomas, 30% of ovarian endometrioid carcinoma, and 40% of low-grade endometrioid adenocarcinomas [8, 9]. The majority of ARID1A mutations are frameshift or nonsense mutations. Since the C-terminal domain of ARID1A is required for assembly into cBAF, all truncating mutations cause loss of function. Notably, ARID1A has a mutually exclusive paralog subunit, ARID1B, which was identified as a major vulnerability in ARID1A-deficient cancer cell lines [10], suggesting that ARID1B is a therapeutic target in ARID1A-deficient cancers. How ARID1A or ARID1B contributes to the biochemical functions of cBAF is still largely unknown.

Here, we describe the reconstitution of all three major BAF complexes, cBAF, PBAF, and ncBAF, and we perform a direct comparison of their biochemical activities. In addition, we reconstitute a variety of cBAF subcomplexes to define roles for several subunits in complex integrity, high-affinity nucleosome binding, and nucleosome sliding activity. Remarkably, we find that ARID1A is required for the histone octamer transfer activity of cBAF, but loss of ARID1A has little effect on other biochemical activities, including ATPase, nucleosome binding, and nucleosome sliding. We also find that ARID1B, a mutually exclusive paralog of ARID1A, can substitute for ARID1A, demonstrating that ARID1B is also required for histone octamer transfer activity. Our study reveals a biochemical function of ARID1A/ARID1B in BAF-mediated chromatin remodeling, suggesting a model in which dysregulation of histone octamer transfer activity of BAF complexes may lead to cancer formation.

## Materials and methods

### Insect cell lines

Sf9 cells (Thermo Fisher Scientific, Waltham, MA, USA) were used for baculovirus production and recombinant protein expression, and grown in ESF921 media (Expression Systems, Davis, CA, USA) at 27°C.

### Expression and purification of human BAF complexes

Human BAF subunit genes were cloned and expressed using the MultiBac baculovirus expression system (Geneva Biotech, Geneva, Switzerland). Genes coding for BRG1 (residue 1–1647 with an N-terminal 3× FLAG-tag and a C-terminal twin Strep-tag), GLTSCR1 (residue 1–1560 with a C-terminal twin Strep-tag), ARID1A, ARID1B, ARID2, *β*-Actin, BAF53a, BCL7a, DPF2, BRD7, BRD9, PBRM1, PHF10, SMARCB1, SMARCC1, SMARCC2, SMARCD1, SMARCE1, and SS18 were synthesized with codon optimization for insect cells (Genewiz, Cambridge, MA, USA). For cBAF reconstitution, 12 subunits were combined in two separate bacmids [One bacmid contains BRG1, *β*-Actin, BAF53a, SMARCB1, SMARCC1, SMARCC2, SMARCD1, and SMARCE1; the other bacmid includes FLAG-tagged ARID1A (or ARID1B), DPF2, BCL7a, and SS18]. For PBAF reconstitution, 13 subunits were combined in one bacmid. For ncBAF reconstitution, 10 subunits were combined in two separate bacmids [one bacmid contains BRG1, *β*-Actin, BAF53a, SMARCC1, SMARCC2, and SMARCD1; the other bacmid includes GLTSCR1, BRD9, BCL7a, and SS18). For BAF8 and BAF10 reconstitutions, 8 and 10 subunits were combined in one bacmid, respectively. Sf9 cells were infected with viruses and cultured for 72 h at 27°C. Cells were harvested and washed with phosphate-buffered saline and then lysed by sonication in lysis buffer [350 mM NaCl, 20 mM HEPES, pH 7.5, 10% glycerol, 0.1% Tween, 1 mM MgCl_2_, 50 μM ZnCl_2_, 1 mM Dithiothreitol (DTT), 1 mM phenylmethylsulphonyl fluoride (PMSF), 1 mM benzamidine, 2 μg/ml Leupeptin, 2 μg/ml Pepstatin A, and 2 μg/ml Chymostatin (Sigma–Aldrich, St. Louis, MO, USA)]. After centrifugation at 40 000 × *g* at 4°C for 20 min, the supernatant was loaded onto a StrepTactin HP column (Cytiva, Marlborough, MA, USA). The column was washed with lysis buffer and Buffer A (150 mM NaCl, 20 mM HEPES, pH 7.5, 10% glycerol, 1 mM MgCl_2_, and 1 mM DTT) and eluted with Buffer A plus 10 mM desthiobiotin (Sigma–Aldrich). The eluted proteins were then subjected to a Q Sepharose column (Cytiva). After washing with Buffer A, the proteins were eluted by a linear salt gradient. The peak fractions were concentrated in Buffer A and flash-frozen in liquid nitrogen. Protein concentration was determined by the densitometry analysis of sodium dodecyl sulfate–polyacrylamide gel electrophoresis (SDS–PAGE) using bovine serum albumin as a standard. The molar concentration of each BAF subcomplex was estimated from the BRG1 subunit concentration. For PBAF and ncBAF, the molar concentration was normalized based on their ATPase activities compared to cBAF with known concentration. Subunit compositions were confirmed by SDS–PAGE and mass spectrometry.

### Nucleosome preparation

Recombinant human histones were expressed in *Escherichia coli* cells and purified as previously described [11, 12]. In brief, expressed histones were purified as inclusion bodies, solubilized in unfolding buffer (7 M guanidinium hydrochloride, 20 mM Tris–HCl, pH 7.5, and 10 mM DTT), and dialyzed against urea dialysis buffer (7 M urea, 10 mM Tris–HCl, pH 8.0, 0.1 M NaCl, 1 mM ethylenediaminetetraacetic acid (EDTA), 0.2 mM PMSF, and 5 mM 2-mercaptoethanol). Samples were injected into tandemly connected Q Sepharose and SP Sepharose columns, and eluted from SP Sepharose by a linear salt gradient. Histone fractions were dialyzed against water with 0.2 mM PMSF and 5 mM 2-mercaptoethanol, and lyophilized. Histone H2A (T120C) were labeled with Cy5, as previously described [13]. The four histones (H2A, H2B, H3, and H4) were mixed in equimolar ratios in unfolding buffer, dialyzed against refolding buffer (2 M NaCl, 10 mM Tris–HCl, pH 7.5, 1 mM EDTA, and 5 mM 2-mercaptoethanol), and purified through a Superdex-200 column. Nucleosomes were reconstituted by mixing octamers with DNA at a 1:1 ratio in HI buffer (2 M NaCl, 10 mM Tris–HCl, pH 7.5, and 5 mM 2-mercaptoethanol), and dialyzed against a linear salt gradient buffer from HI to LO buffer (50 mM NaCl, 10 mM Tris–HCl, pH 7.5, and 5 mM 2-mercaptoethanol) for 20 h.

### Histone octamer transfer assays

For histone octamer transfer assays, Cy5-labeled histone H2A-containing mononucleosomes were reconstituted by salt dialysis onto 197-bp DNA fragments containing the 601 nucleosome-positioning sequence in the center of the DNA fragment (25N25). Mononucleosomes (15 nM) were incubated with BAF (60 nM), 0N0 DNA fragments (10 nM), and 2 mM ATP in Buffer B (25 mM NaCl, 25 mM HEPES, pH 7.5, 5 mM MgCl_2_, and 1 mM DTT) at 30°C for 60 min. The reactions were initiated by addition of ATP. At each time point, the reactions were quenched with 5% glycerol and 0.1 mg/ml salmon sperm DNA, incubated for 5 min at 30°C, and resolved on 5% Native-PAGE in 0.5× Tris-Borate-EDTA (TBE). Gels were scanned with Cy5 using a Typhoon biomolecular imager (GE Healthcare, Chicago, IL, USA). To modify 25N25 DNA fragments with biotin at both ends, PCR was performed using biotinylated primers for both ends. The fraction of 0N0 nucleosome product was quantified and plotted over time. Transfer rate obtained from the plot was normalized to a rate of the wild-type (WT) cBAF.

For FRET assays, Cy5-labeled histone H2A-containing mononucleosomes were reconstituted by salt dialysis onto 25N25 DNA fragments. Mononucleosomes (15 nM) were incubated with BAF (60 nM), Cy3-labeled 0N0 DNA fragments (10 nM), and 2 mM ATP in Buffer B at 30°C for 60 min. The FRET signal was monitored using a Tecan Spark microplate reader (Tecan, Switzerland) with excitation at 530 nm and emission at 670 nm.

### Nucleosome sliding assays

*HhaI* restriction enzyme accessibility assays were performed as described [14]. Mononucleosomes were reconstituted by salt dialysis onto Cy5-labeled 25N25 DNA fragments. Mononucleosomes (15 nM) were incubated with BAF (2, 3, or 16 nM), 4 units/μl *HhaI*, and 2 mM ATP in buffer B at 30°C. At each time point, the reactions were stopped by addition of STOP buffer (10 mM HEPES, pH 7.5, 40 mM EDTA, 0.6% SDS, 5% glycerol, 0.1 mg/ml Proteinase K), incubated for 20 min at 50°C, and resolved on 6% Native-PAGE in 0.5× TBE. Gels were scanned with Cy5 using a Typhoon biomolecular imager. The amounts of slid nucleosomes were calculated from the fraction of cut/uncut DNA and plotted over time. Sliding rate (s^−1^) was obtained from an initial linear slop of the plot divided by BAF concentration.

### Fluorescence polarization assays

Fluorescence polarization (FP) assays were performed as described [15]. Various concentrations of BAF were incubated with 10 nM Cy5-labeled mononucleosomes in Buffer B, except for 60 mM NaCl. FP were measured using a Tecan Spark microplate reader. All binding curves were fit to the quadratic binding equation:


\begin{equation*}
{\mathrm{Y}} = {{B}} + \left( {{{T}} - {{B}}} \right)*\left( {{{K}} + {{F}} + {{X - {\rm sqrt}}}\left( {{\mathrm{sqr}}\left( {{{K}} + {{F}} + {{X}}} \right) - \left( {4*{{F}}*{{X}}} \right)} \right)} \right)/2*{{F}},
\end{equation*}

where *B* and *T* are the minimum and maximum FP signals, respectively, *K* is the apparent dissociation constant, *F* is the nucleosome concentration, and *X* is the concentration of BAF.

### ATPase assays

ATPase assays were performed as previously described [16]. ATP hydrolysis was monitored using NAD/NADH-coupled ATP regeneration system with lactate dehydrogenase (LDH) and pyruvate kinase (PK). Fifty nanomolar BAF was incubated with 10% PK/LDH mixture (Sigma), 1 mM phosphoenolpyruvate, 1 mM NADH, and 1 mM ATP in Buffer B. One hundred nanomolar 25N25 DNA fragment was used as a substrate. NADH absorbance was measured at 340 nm using a Tecan Spark microplate reader. ATPase activity was obtained from an initial linear rate subtracted from background signal (without BAF).

### Histone eviction assays

For histone eviction assays, mononucleosomes were reconstituted by salt dialysis onto Cy5-labeled 25N25 DNA fragments. Mononucleosomes (15 nM) were incubated with BAF (60 nM) and 2 mM ATP in Buffer B at 30°C for 90 min. The reactions were initiated by addition of ATP. At each time point, the reactions were quenched with 5% glycerol and 0.1 mg/ml salmon sperm DNA, incubated for 5 min at 30°C, and resolved on 5% Native-PAGE in 0.5× TBE. Gels were scanned with Cy5 using a Typhoon imager. The fraction of free 25N25 DNA was quantified and plotted over time.

### Electrophoretic mobility shift assays for nucleosome binding and deposition

For nucleosome-binding assays, Cy5-labeled histone H2A-containing mononucleosomes were reconstituted by salt dialysis onto 0N0 DNA fragments. Mononucleosomes (15 nM) were incubated with various concentrations of IDR2 or ARID + IDR2 in Buffer B for 15 min at room temperature. The reactions were resolved on 5% Native-PAGE in 0.25× TBE. Gels were scanned using a Typhoon imager.

For nucleosome deposition assays, Cy5-labeled histone H2A-containing octamers and 0N0 DNA fragments were mixed in equimolar ratios (each 100 nM) with various concentrations of IDR2 in Buffer B for 15 min at room temperature. The reactions were resolved on 5% Native-PAGE in 0.5× TBE. Gels were scanned using a Typhoon imager.

## Results

### Histone octamer transfer activity is conserved in all three BAF complexes

**Figure 1.**
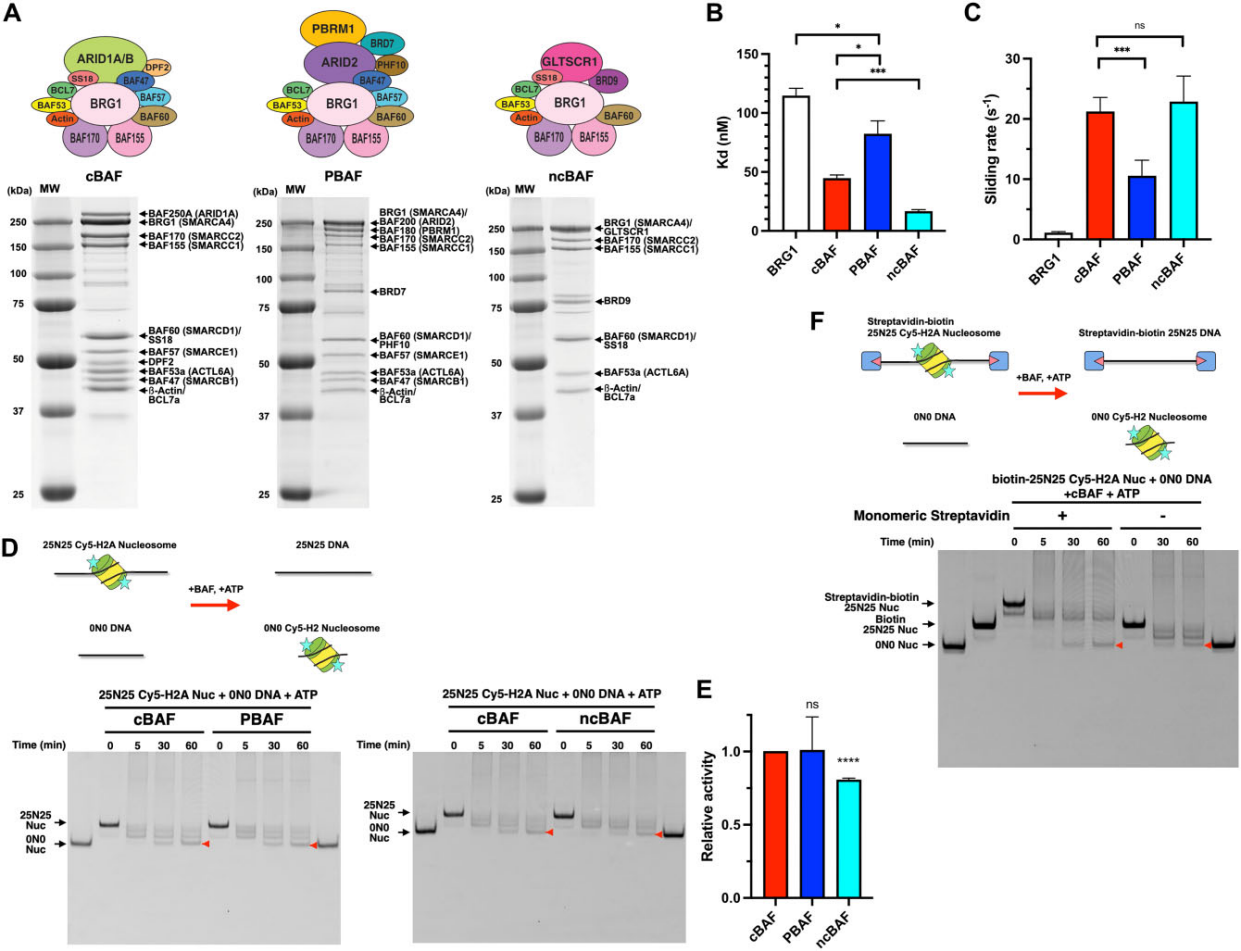
Histone octamer transfer activity is conserved in all three BAF complexes. (**A**) Coomassie-staining SDS–PAGE gels of purified cBAF (left panel), PBAF (middle panel), and ncBAF (right panel). (**B**) Nucleosome-binding affinity of cBAF, PBAF, and ncBAF. (**C**) Nucleosome sliding activity of cBAF, PBAF, and ncBAF. (**D**) Histone octamer transfer activity of cBAF, PBAF, and ncBAF. Schematic of histone octamer transfer assay (top panel). Representative Cy5-scanned native gels of histone octamer transfer assay (bottom panels). Red arrow indicates 0N0 nucleosomes produced by octamer transfer. (**E**) Quantification of histone octamer transfer activity. Each activity was normalized to cBAF activity. (**F**) Representative Cy5-scanned native gel of histone octamer transfer assay using streptavidin-biotinylated nucleosomes. For panels (B), (C), and (E), each error bar represents the standard error from at least three independent experiments using at least two independent BAF preparations. ****P< .0001; ***P< .001; *P< .05; ns, not significant.

**Figure 2.**
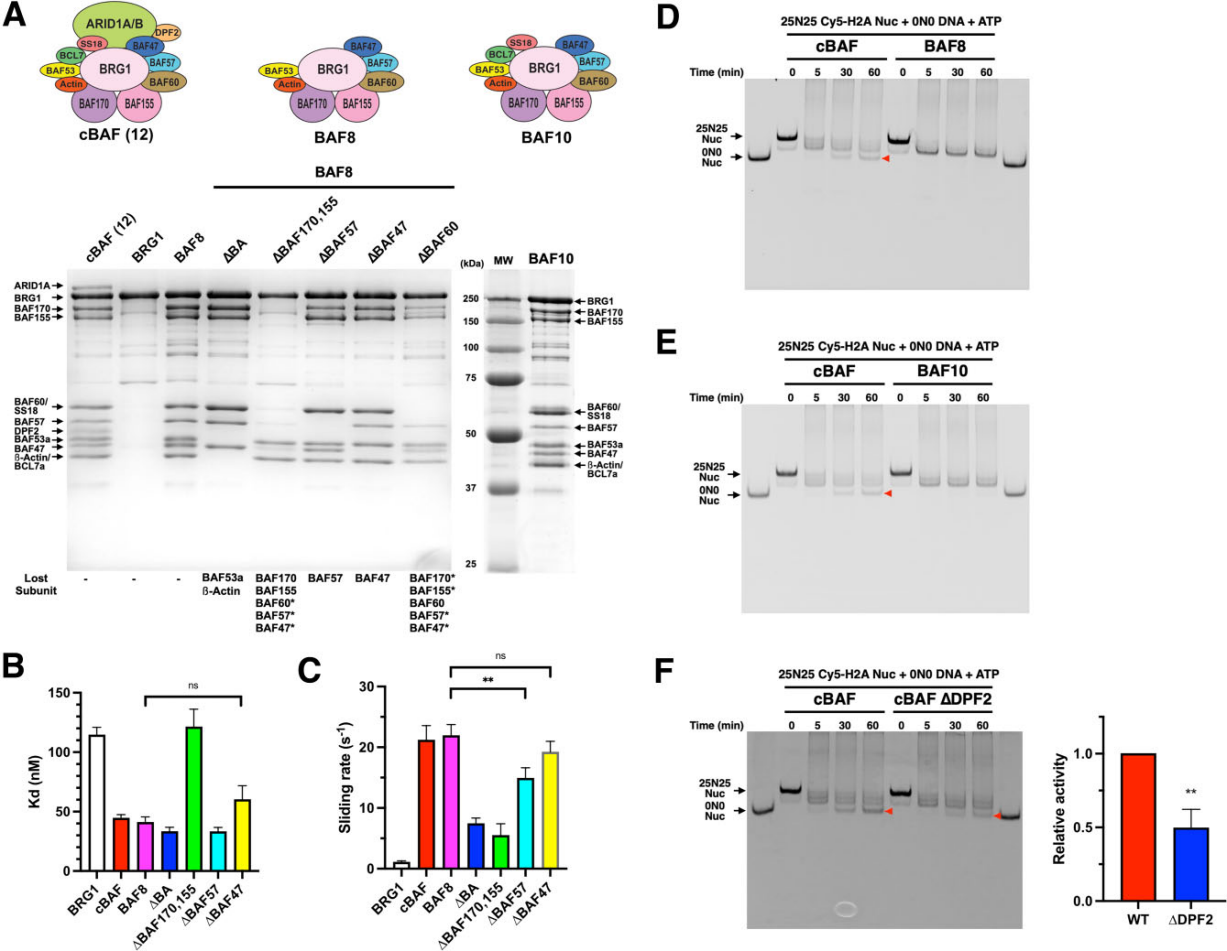
The core 8-subunit BAF complex lacks histone octamer transfer activity. (**A**) Coomassie-staining SDS–PAGE gels of purified BAF subcomplexes. (**B**) Nucleosome-binding affinity of BAF8 subcomplexes. (**C**) Nucleosome sliding activity of BAF8 subcomplexes. (**D**) Representative Cy5-scanned native gel of histone octamer transfer assay for BAF8. Red arrow indicates 0N0 nucleosomes produced by octamer transfer. (**E**) Representative Cy5-scanned native gel of histone octamer transfer assay for BAF10. (**F**) Representative Cy5-scanned native gel (left panel) and quantification (right panel) of histone octamer transfer assay for cBAF ΔDPF2 complex. The activity was normalized to cBAF WT activity. For panels (B), (C), and (F), each error bar represents the standard error from at least three independent experiments using at least two independent BAF preparations. **P< .01; ns, not significant.

To investigate the detailed biochemical properties of the three major BAF complexes, we reconstituted human cBAF, PBAF, and ncBAF complexes with the full-length, recombinant subunits using a baculoviral expression system and Strep-tag affinity purification. The subunit compositions were confirmed by SDS–PAGE (Fig. 1A) and mass-spectrometry (Supplementary Tables S1 and S2). The individual BRG1 ATPase subunit was also purified so that its activity could be compared to the intact BAF complexes (Fig. 2A).

To quantify the ability of each BAF complex to bind its nucleosomal substrate, a FP assay was employed (Fig. 1B and Supplementary Fig. S1A). Recombinant nucleosomes were reconstituted with human histone octamers that contained a Cy5-labeled histone H2A and a 197-bp DNA template that harbored a “601” nucleosome positioning sequence. As expected, the isolated BRG1 subunit bound to nucleosomes weaker, in comparison to the BAF complexes, with a Kd of 115 nM. The ncBAF complex exhibited the highest affinity for nucleosomes, with a Kd of 17 nM, followed by cBAF at 45 nM and PBAF at 82 nM.

We then measured the ATP-dependent nucleosome sliding activity of each remodeler, using a quantitative restriction enzyme accessibility assay. Each BAF complex catalyzed the sliding of nucleosomes at rates that were at least 10-fold higher than the isolated BRG1 subunit (Fig. 1C and Supplementary Figs S1B and S2B). Each BAF complex had comparable sliding rates, though the cBAF and ncBAF complexes were ∼2× more effective than PBAF (Fig. 1C and Supplementary Fig. S1B). These small rate differences may be due to their differing binding affinities, as these assays were performed with remodeler concentrations below Kd. However, the decreased sliding rate of PBAF, as compared to the activities of cBAF and ncBAF, is consistent with previously published results that used endogenous BAF complexes and a modified nucleosome library [17].

Whereas most remodeling enzymes can slide nucleosomes in *cis*, SWI/SNF complexes are also able to transfer an entire histone octamer from one nucleosome to a different DNA fragment. To measure this histone octamer transfer activity, BAF complexes were incubated with a Cy5-labeled histone H2A-containing 25N25 nucleosome and a 147-bp DNA (0N0), and the reaction was initiated by addition of ATP (Fig. 1D, top). The histone octamer transfer activity of BAF led to the ATP-dependent formation of a Cy5-labeled 0N0 nucleosome, monitored by Native-PAGE and a Cy5 scan (see red arrowhead). Note that formation of this product requires addition of the 0N0 DNA (Supplementary Fig. S1C). All three BAF complexes showed comparable histone octamer transfer activity (Fig. 1D, bottom, and E). Notably, under these assay conditions, almost all nucleosomes were re-positioned from the original center position to the end of the 25N25 DNA fragment within 5 min. We note that two additional, faster migrating species were also observed. The faster migrating, major band is the end-position nucleosome, and the slower migrating, weak band may be a common, intermediate slid nucleosome that was also observed in all other experiments of this study and by others [5]. In contrast, the product of the histone octamer transfer reaction, the 0N0 nucleosome, accumulated at 30–60 min, suggesting that histone octamer transfer may occur either subsequent to completion of the sliding reaction or that these two reactions may not be coupled.

One possibility is that histone octamer transfer activity involves the sliding of histone octamers completely “off the ends” of the original DNA fragment. To test this idea, both ends of the 25N25 DNA fragment were modified with biotin so that streptavidin could be used to block each DNA end, as previously demonstrated [18, 19] (Fig. 1F, top). Cy5-H2A-containing nucleosomes were reconstituted with the biotinylated 25N25 DNA fragments, and the addition of monomeric streptavidin led to the expected mobility shift of the biotin-25N25 nucleosomes on Native-PAGE (compare lanes 2 and 3 in Fig. 1F, bottom). We then compared the histone octamer transfer activity of cBAF in the presence or absence of monomeric streptavidin (compare lanes 6 and 9 in Fig. 1F). Importantly, the addition of streptavidin did not affect the histone transfer activity of cBAF, suggesting that a free DNA end is not required for histone octamer transfer.

It is also possible that BAF complexes do not transfer histone octamers, but rather BAF can only evict histone octamers and that these dissociated histones are then passively assembled onto free DNA. To assess the histone eviction activity of BAF complexes, nucleosomes were reconstituted onto a Cy5-labeled 25N25 DNA fragment and incubated with BAF complexes in the absence of 0N0 DNA. In this assay, histone octamer eviction leads to an increased amount of free, Cy5-labeled 25N25 DNA, monitored by Native-PAGE and a Cy5 scan. All three BAF complexes increased the fraction of free 25N25 DNA by only ∼5% within 5 min after ATP addition, and this level remained unchanged for an extended time course to 90 min (Supplementary Fig. S1D). These results suggest that a limited amount of histone octamer eviction may be coupled to nucleosome sliding, unlike histone octamer transfer. Importantly, given that the fraction of the transferred nucleosomes to 0N0 DNA fragments in the histone octamer transfer assays was estimated to be 20%–25% after 60 min time course, the results suggest that histone octamer eviction is not a major contributor to histone octamer transfer activity.

### The core eight-subunit BAF complex lacks histone octamer transfer activity

To further investigate the assembly and function of BAF complexes, we reconstituted a BAF subcomplex with eight core subunits shared between cBAF and PBAF complexes (BRG1, BAF170, BAF155, BAF60, BAF57, BAF47, BAF53a, and *ß*-Actin), which we refer to here as “BAF8” (Fig. 2A, BAF8). Various subunits were also omitted from the BAF8 reconstitution, leading to a set of smaller subcomplexes (Fig. 2A). Previous glycerol gradient purification analyses of endogenous BAF complexes suggested that there may be a BAF core module containing five subunits (BAF170, BAF155, BAF60, BAF57, and BAF47) that stably exists as a precursor [20]. When BAF53a and *ß*-Actin were removed from BAF8, the assembly of other subunits with BRG1 was not impacted (Fig. 2A, ΔBA). Since endogenous *ß*-Actin exists abundantly, this result suggests that BAF53a might be required for the association of *ß*-Actin with the BAF complex. Since BAF170 and BAF155 are interchangeable [5], both were eliminated from BAF8, leading to the loss of many subunits, with the exception of *ß*-Actin and BAF53a (Fig. 2A,ΔBAF170, 155). Removal of either BAF57 or BAF47 had no effect on the association of the remaining subunits with the complex (Fig. 2A, ΔBAF57 and ΔBAF47), whereas elimination of BAF60 resulted in reduced amounts of all Base subunits (BAF170, BAF155, BAF57, and BAF47) (Fig. 2A, ΔBAF60). These results suggest that (i) the ARP module and the Base module interact independently with BRG1 and (ii) BAF170, BAF155, and BAF60 are required for the overall integrity of the Base module, consistent with previous work with endogenous complexes [20].

We next measured the nucleosome-binding affinity and nucleosome sliding activity of BAF8 and BAF8 subcomplexes (Fig. 2B and C, and Supplementary Fig. S2A and B). The cBAF and BAF8 complexes were nearly identical in these assays, suggesting that the four other subunits of cBAF (BCL7, SS18, ARID1A, and DPF2) do not contribute significantly to nucleosome binding or nucleosome sliding activities. Likewise, the ΔBAF57 and ΔBAF47 subcomplexes had similar nucleosome sliding rates and nucleosome-binding affinities as compared to the BAF8 and cBAF complexes, indicating that BAF57 and BAF47 also do not contribute significantly to these activities, despite the fact that BAF47 is known to make contact with the nucleosomal acidic patch within the cBAF-nucleosome and PBAF-nucleosome complexes [5, 6]. In contrast, loss of the ARP module (ΔBA) reduced the nucleosome sliding rate, though nucleosome-binding affinity was unaffected. Strikingly, loss of the Base module (ΔBAF170, 155) reduced both nucleosome sliding activity and nucleosome binding, with binding affinity similar to that of the isolated BRG1 subunit. These results suggest that (i) the ARP module is required for proper nucleosome sliding but not for nucleosome binding and (ii) the Base module subunits, except for BAF57 and BAF47, are required for proper nucleosome sliding and nucleosome binding.

### ARID1A is required for histone octamer transfer activity of cBAF

**Figure 3.**
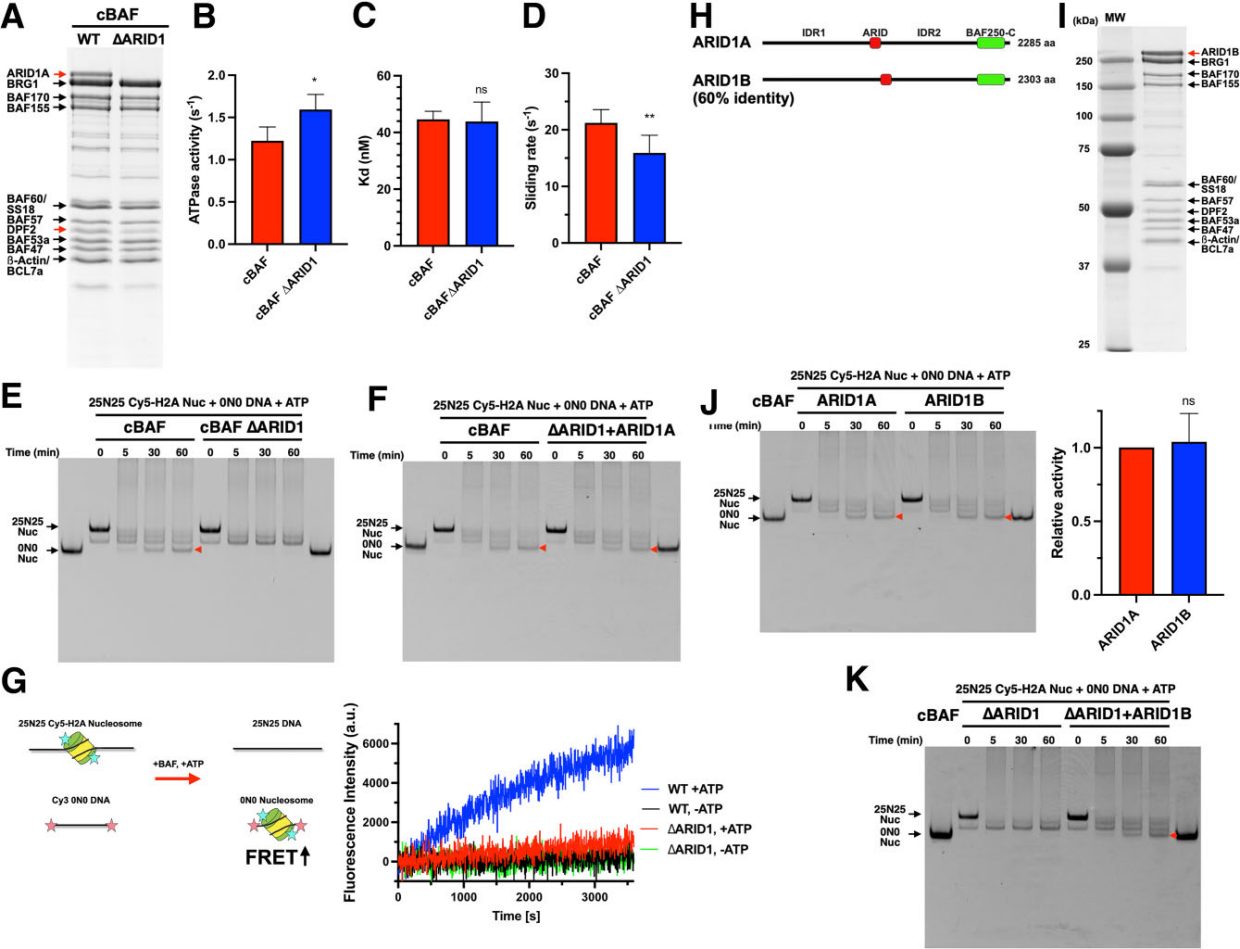
ARID1A/ARID1B are required for histone octamer transfer activity. (**A**) Coomassie-staining SDS–PAGE gel of purified cBAF ΔARID1 complex. (**B**) ATPase activity of cBAF ΔARID1. (**C**) Nucleosome-binding affinity of cBAF ΔARID1. (**D**) Nucleosome sliding activity of cBAF ΔARID1. (**E**) Representative Cy5-scanned native gel of histone octamer transfer assay for cBAF ΔARID1. Red arrow indicates 0N0 nucleosomes produced by octamer transfer. (**F**) Representative Cy5-scanned native gel of histone octamer transfer assay for cBAF ΔARID1 after adding back ARID1A. (**G**) FRET-based assay for monitoring histone octamer transfer activity. Schematic of the FRET-based assay (left panel). Representative FRET signal changes of cBAF WT and cBAF ΔARID1 in the presence or absence of ATP (right panel). (**H**) Domain structures of ARID1A and ARID1B. (**I**) Coomassie-staining SDS–PAGE gel of purified ARID1B-containing cBAF. (**J**) Representative Cy5-scanned native gel of histone octamer transfer assay for ARID1B-containing cBAF. The activity was normalized to ARID1A-containing cBAF activity. (**K**) Representative Cy5-scanned native gel of histone octamer transfer assay for cBAF ΔARID1 after adding back ARID1B. For panels (B), (C), (D), and (J), each error bar represents the standard error from at least three independent experiments using at least two independent BAF preparations. **P< .01; *P< .05; ns, not significant.

Finally, we measured the histone octamer transfer activity of BAF8. Surprisingly, BAF8 did not exhibit detectable histone octamer transfer activity (Fig. 2D), suggesting that the four subunits of cBAF that are missing from BAF8 (BCL7a, SS18, ARID1A, and DPF2) are required for histone octamer transfer activity. To identify which subunit(s) is required for histone octamer transfer activity, we added the BCL7a and SS18 subunits to BAF8, leading to a BAF10 subcomplex (Fig. 2A). However, BAF10 remained inactive in the histone octamer transfer assay (Fig. 2E). Reconstitution of a cBAF complex that only lacked the DPF2 subunit had no effect on the overall integrity of cBAF (Supplementary Fig. S2C), but reduced histone octamer transfer activity (Fig. 2F), suggesting that DPF2 contributes to histone octamer transfer activity, but is not essential. Finally, we assembled a cBAF complex that lacks ARID1A (ΔARID1 complex). Removal of ARID1A did not grossly change the composition of BAF (Fig. 3A), except for reduced level of DPF2, consistent with the known interaction between ARID1A and DPF2 [5]. Whereas loss of ARID1A led only to small defects in ATPase, nucleosome binding, and nucleosome sliding activities (Fig. 3B–D, and Supplementary Fig. S3A and B), the ΔARID1 complex was inactive in the histone octamer transfer assay (Fig. 3E). Adding back purified, recombinant ARID1A (Supplementary Fig. S3C) to the ΔARID1 complex restored histone octamer transfer activity (Fig. 3F), confirming that ARID1A is required for the histone octamer transfer activity of cBAF.

In order to monitor histone octamer transfer activity by an alternative method, we developed a FRET-based assay. In this assay, BAF complexes were incubated with Cy5-labeled histone H2A nucleosomes and a Cy3-labeled DNA fragment. Transfer of the Cy5-labeled octamer (donor fluorophore) to the Cy3-labeled DNA (acceptor fluorophore) is expected to lead to an increase in FRET (Fig. 3G, left). As expected, in the presence of cBAF, an ATP-dependent increase in FRET signal was observed over time (Fig. 3G, right, and blue line). On the other hand, the ΔARID1 complex was nearly inactive in this FRET-based histone octamer transfer assay (Fig. 3G, right, and red line). Taking together, these findings demonstrate that ARID1A is required for the histone octamer transfer activity of cBAF.

### ARID1B is also required for the histone octamer transfer activity of cBAF

ARID1B is a mutually exclusive paralog subunit of ARID1A with 60% identity (Fig. 3H). Loss of ARID1B is synthetically lethal in ARID1A-deficient cancer cells, suggesting that ARID1B is a therapeutic target in ARID1A-deficient cancers. To test whether ARID1B can promote the histone octamer transfer activity of cBAF, we reconstituted an ARID1B-containing cBAF complex. The subunit composition of this cBAF complex was the same as that of ARID1A-containing cBAF (Fig. 3I), and nucleosome sliding activity also appeared to be similar (Fig. 3J). Furthermore, the histone octamer transfer assay revealed that the ARID1B-containing complex exhibited comparable histone octamer transfer activity to the ARID1A-containing complex (Fig. 3J), demonstrating that ARID1B can also support the histone octamer transfer activity of cBAF. Furthermore, we purified recombinant ARID1B (Supplementary Fig. S3D) and added it back to the reaction in the presence of cBAF ΔARID1 complex. Similar to ARID1A, add-back of ARID1B to the ΔARID1 complex restored histone octamer transfer activity (Fig. 3K). Taking together, these results revealed that ARID1A and ARID1B are key subunits required for the histone octamer transfer activity of cBAF.

### A part of IDR2 is required for histone octamer transfer activity of cBAF

**Figure 4.**
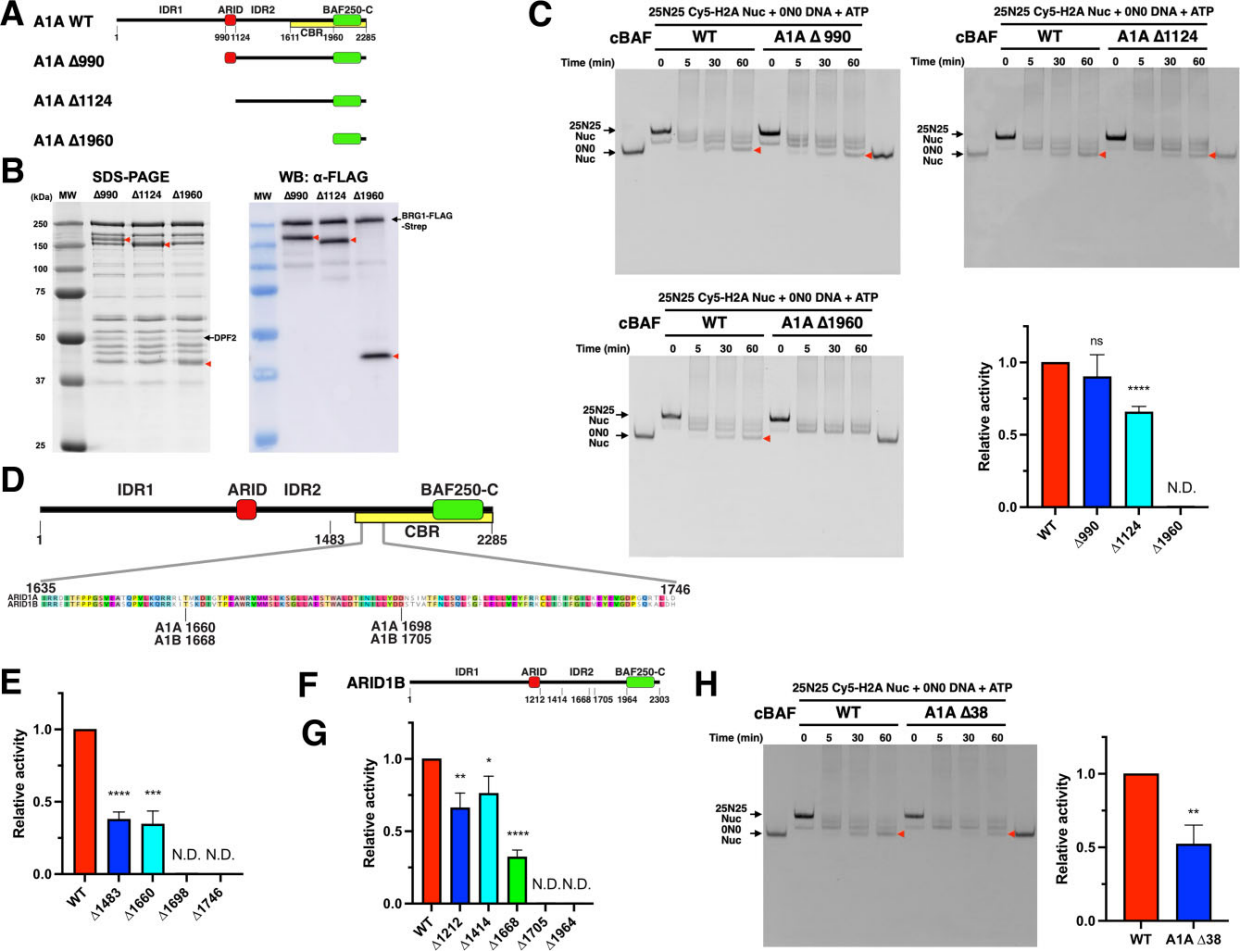
A part of IDR2 is required for histone transfer activity of cBAF. (**A**) Domain structure of ARID1A truncation constructs. CBR, core-binding region [21]. (**B**) Coomassie-staining SDS–PAGE gel (left panel) and western blot image (right panel) of cBAF complexes containing ARID1A truncations. All ARID1A truncations and BRG1 contain 3× FLAG-tag at the N-terminus. (**C**) Representative Cy5-scanned native gel of histone octamer transfer assay for cBAF complexes containing ARID1A truncations. Red arrow indicates 0N0 nucleosomes produced by octamer transfer. Each activity was normalized to WT. (**D**) Sequence alignment of a region that is required for histone octamer transfer activity. (**E**) Quantification of histone octamer transfer activity of cBAF complexes containing ARID1A truncations. Each activity was normalized to WT. (**F**) Domain structure of ARID1B truncation constructs. (**G**) Quantification of histone octamer transfer activity of cBAF complexes containing ARID1B truncations. Each activity was normalized to WT. (**H**) Representative Cy5-scanned native gel and quantification of histone octamer transfer activity of cBAF complex containing ARID1A Δ38. The activity was normalized to WT. For panels (C), (E), (G), and (H), each error bar represents the standard error from at least three independent experiments using at least two independent BAF preparations. ****P< .0001; ***P< .001; **P< .01; *P< .05; ns, not significant; and N.D., not detected.

Previous studies have identified four distinct domains within ARID1A (Fig. 4A). The C-terminus of ARID1A is highly conserved between the two ARID1 paralogs, and this region contains seven ARM domains that are key for assembly of ARID1 subunits into the cBAF complex. There are two intrinsically disordered regions (IDR1 and IDR2) that can form phase condensates *in vitro* and contribute to ARID1A function *in vivo* [21]. Finally, there is an ARID domain that confers DNA binding to AT-rich DNA sequences (Fig. 4A). To identify a region in ARID1A required for the histone octamer transfer activity of cBAF, we initially expressed and reconstituted cBAF complexes with three different N-terminal truncation derivatives of ARID1A (Fig. 4A). A1A Δ990 removes IDR1, A1A Δ1124 removes both the IDR1 and the ARID domain, and A1A Δ1960 removes IDR1, ARID, and IDR2 (Fig. 4A). Each of these ARID1A truncation derivatives assembled into cBAF complexes, as confirmed by SDS–PAGE and western blot (Fig. 4B). It should be noted that the levels of DPF2 were reduced in the cBAF complex that contains the largest truncation, A1A Δ1960 (Fig. 4B), consistent with structural studies showing that DPF2 interacts with a segment of the IDR2 domain [5].

Histone octamer transfer activity was measured for the cBAF complexes that harbor different ARID1A truncations. Removal of the IDR1 domain (A1A Δ990) had no significant impact on histone octamer transfer activity, whereas the additional removal of the ARID domain (A1A Δ1124) showed 30%–40% reduction in activity (Fig. 4C). Finally, removal of IDR1, IDR2, and the ARID domain (A1A Δ1960) eliminated histone octamer transfer activity (Fig. 4C). These results indicated that ARID1A residues between 1124 and 1960 are required for histone transfer activity, though the ARID domain also appears to contribute.

Four additional ARID1A truncation derivatives were reconstituted into cBAF complexes, defining an ∼40 amino acid region that was required for optimal histone octamer transfer activity (compare A1A Δ1660 and A1A Δ1698; Fig. 4D and E, and Supplementary Fig. S4A and B). This region is highly conserved between ARID1A and ARID1B, and it is one of a few short, structured domains within IDR2 (Fig. 4D). Note that the A1A 1698 deletion eliminates histone octamer transfer activity without significant loss of the DPF2 subunit from cBAF (Supplementary Fig. S4A). We also generated various truncation constructs of ARID1B, and then reconstituted cBAF with each of these ARID1B variants. As for ARID1A, a similar, conserved region within ARID1B (a.a.1668–1705) was required for the histone octamer transfer activity of cBAF (Fig. 4F and G, and Supplementary Fig. S4C and D). Finally, an internal deletion of ARID1A was constructed that removed only the 38 amino acid conserved domain (a.a. 1661-1698). The cBAF complex harboring ARID1A Δ38 retained only 52% of the WT level of histone octamer transfer activity, confirming the key role of this region (Fig. 4H and Supplementary Fig. S4E). These data also reinforce the view that other N-terminal domains of ARID1A, notably the ARID domain, also play a role in histone octamer transfer activity.

One possible mechanism of how this IDR2 region mediates the histone octamer transfer activity of cBAF is that the IDR2 region binds to a nucleosome and mediates histone octamer transfer. To test this possibility, we expressed and purified the entire IDR2 region (Supplementary Fig. S4F) to access its nucleosome binding activity. Electrophoretic mobility shift assays did not detect nucleosome binding activity for IDR2 (Supplementary Fig. S4G). In contrast, the protein fragment containing both the IDR2 and the ARID domain bound to nucleosomes, likely through the DNA binding of the ARID domain (Supplementary Fig. S4H).

Another possibility is that the IDR2 region has a nucleosome deposition activity like histone chaperones. To test this, the purified IDR2 region was incubated with Cy5-labeled histone octamers and free 0N0 DNA fragments. The formation of 0N0 nucleosomes was not observed, suggesting that the IDR2 region does not have nucleosome deposition activity (Supplementary Fig. S4I). These data also confirmed that free histone octamers do not passively form nucleosomes onto DNA fragments under our assay conditions.

## Discussion

Despite the clear importance of BAF complexes in development, transcriptional regulation, and disease prevention, distinct biological and biochemical functions for the three major forms of BAF complexes (cBAF, PBAF, and ncBAF) have remained largely unexplored. Recently a mononucleosome library screening approach found that cBAF, PBAF, and ncBAF complexes recognized distinct histone modification patterns [17]. Here, we reconstituted the cBAF, PBAF, and ncBAF complexes and directly compared their biochemical activities. Each complex showed distinct activities, with cBAF and ncBAF showing higher affinity nucleosome binding, compared to PBAF. Note that PBRM1, a PBAF-specific subunit, contains multiple Bromo domains, and thus it is likely that histone acetylation may impact nucleosome-binding affinity and/or other biochemical activities. Perhaps not surprisingly, all three multi-subunit complexes were more active than the isolated BRG1 ATPase subunit. The trend of these biochemical properties is consistent with previous studies with endogenous BAF complexes [17].

CryoEM structures of cBAF and PBAF reveal that the BAF47 subunit interacts with one face of the nucleosome, sandwiching the nucleosome between this contact and the BRG1 ATPase. Surprisingly, our biochemical analysis of BAF8 subcomplexes showed that the loss of BAF47 had only a mild impact on nucleosome-binding affinity and nucleosome sliding activity, suggesting that nucleosome recognition by BAF47 may not play a major role in these remodeling activities. Notably, the ncBAF complex does not contain the BAF47 subunit, though it remains a possibility that GLTSCR1, an ncBAF-specific subunit, might substitute for BAF47, contributing to the increased nucleosomal binding that we observed for ncBAF compared to cBAF and PBAF.

In addition to reconstituting intact BAF complexes, we also probed how different subunits impact BAF assembly and activity. Largely consistent with previous work, we confirmed that the BAF170/BAF155 subunits are key for assembly of cBAF. Furthermore, our biochemical analysis of BAF8 subcomplexes suggested that the Base module is important for both nucleosome binding and nucleosome sliding activities, whereas the ARP module is required only for robust levels of nucleosome sliding, consistent with the notion that the ARP module is involved in “coupling” of the ATPase and sliding activities [22]. The most striking result from this work was our finding that ARID1A was required for the histone octamer transfer activity of cBAF. Loss of ARID1A from cBAF had little impact on nucleosome-binding affinity or nucleosome sliding activity, indicating that it provides a unique function. Since both PBAF and ncBAF complexes also exhibit histone octamer transfer activity, our data suggest that complex-specific subunits, such as ARID2 and GLTSCR1, may play a similar role, although the contribution of individual ncBAF/PBAF-specific subunits to transfer activity remains an open question.

The mechanism by which ARID1A/ARID1B mediates the histone octamer transfer activity of cBAF is unclear. Our analysis of a series of ARID1A truncations suggests that a 38-amino acid domain of ARID1A is required for the histone octamer transfer activity of cBAF. Interestingly, in the cryoEM structure of cBAF, this domain is comprised of two alpha-helixes at the end of the ARM-like structure of the C-terminal domain of ARID1A. C-terminal to this domain, the ARID1A polypeptide extends to the proximity of the DPF2 subunit, returning to form the remainder of ARM-like structure. One possibility is that this new functional domain mediates the transfer of histone octamers directly through an association with either histones or DNA. Consistent with this model, our deletion analyses indicates that this domain may function in concert with the ARID DNA binding domain. Given that this region is near the ARM domain that interacts with the BRG1 scaffold, an alternative view is that deletion of this domain may alter the conformation of the Base module, which may be key for mediating histone octamer transfer activity.

Recent genome-wide localization studies indicate that BAF complexes occupy nearly 41 000 genomic sites that include TSS-proximal (e.g. promoters) and TSS-distal (e.g. enhancers) locations [21]. These localization patterns are consistent with the established role for BAF complexes in broadly controlling enhancer function [23–26]. Loss of ARID1 subunits disrupts the recruitment of BAF complexes to ∼10 000 of these genomic sites, primarily impacting TSS-distal locations [21]. Furthermore, loss of ARID1 subunits reduces chromatin accessibility at many sites [21, 24, 27, 28]. ARID1A domain mapping studies indicated that N-terminal regions of ARID1A play key roles in BAF targeting and recruitment [21]. Although much emphasis has been placed on the IDR1 region, which drives phase condensation *in vitro* and *in vivo*, regions that we identify here as important for histone octamer transfer activity are also encompassed by deletions that impair BAF targeting and *in vivo* activity [21]. Given that BAF targeting is also associated with increased chromatin accessibility, we favor a model in which histone octamer transfer activity plays a dominant role in facilitating the formation of open chromatin domains.

## Supplementary Material

gkaf958_Supplemental_File

## Data Availability

The data are available from the corresponding author upon reasonable request.
